# High PrEP uptake, adherence, persistence and effectiveness outcomes among young Thai men and transgender women who sell sex in Bangkok and Pattaya, Thailand: findings from the open-label combination HIV prevention effectiveness (COPE) study

**DOI:** 10.1016/j.lansea.2023.100217

**Published:** 2023-05-25

**Authors:** Brian W. Weir, Andrea L. Wirtz, Tareerat Chemnasiri, Stefan D. Baral, Michele Decker, Chen Dun, Sandra Hsu Hnin Mon, Chaiwat Ungsedhapand, Eileen F. Dunne, Joseph Woodring, Sarika Pattanasin, Wichuda Sukwicha, Michael C. Thigpen, Anchalee Varangrat, Anchalee Warapornmongkholkul, Siobhan O'Connor, Julie P. Ngo, Noor Qaragholi, Haley I. Sisel, Jasmine M. Truong, Surang Janyam, Danai Linjongrat, Somchai Sriplienchan, Pachara Sirivongrangson, James F. Rooney, Patrick Sullivan, Boosbun Chua-Intra, Andrew C. Hickey, Chris Beyrer, COPE Study Team, Chris Beyrer, Andrea Wirtz, Brian Weir, Stefan Baral, Michele Decker, Sandra Hsu Hnin Mon, James Case, Chen Dun, Jasmine Truong, Noor Qaragholi, Julie Ngo, Haley Sisel, Pachara Sirivongrangson, Boosbun Chua-Intra, Anupong Chitwarakorn, Wasin Matsee, Pratakpong Wongkiti, Chidanan Krasan, Anchana Chainuwong, Nauwarat Imlimtharn, Potcharawan Reansoi, Teeraparp Watanatanyaporn, Jarupa Nuamlert, Supannikar Namwong, Jutarat Phetnark, Wachirawit Supasa, Siriporn Sueayot, Andrew Hickey, Michael Thigpen, Eileen Dunne, Joseph Woodring, Christie Vu, Siobhan O'Connor, Patrick Flaherty, Timothy Holtz, Tareerat Chemnasiri, Anchalee Varangrat, Anchalee Warapornmongkholkul, Anekpong Chanthaweesirirat, Warunee Thienkrua, Wichuda Sukwicha, Pitthaya Disprayoon, Kanjana Kamkong, Dararat Worrajittanon, Supawadee Na-Pompet, Chonlanot Sariwatta, Patnaree Oungprasertgul, Phanurassamee Sittidech, Jirawat Suksamosorn, Kesinee Sujina, Chaiwat Ungsedhapand, Wannee Chonwattana, Nichnawee Kamchaithep, Sarika Pattanasin, Nongkran Tatakham, Pikunchai Luechai, Philip Mock, Betsy Cadwell, Ram Shrestha, Baranee Balmongkol, Boonyos Raengsakulrach, Wanna Leelawiwat, Wanna Suwannaphan, Achara Sriinsut, Punneeporn Wasinrapee, Pornchanok Chanathalay, Nutthawoot Promda, Santi Winaitham, Oranuch Kongpechsatit, Kusuma Auethavornanan, Jaray Tongtoyai, Pairote Tararut, Atitaya McNamara, Famui Mueanpai, Natthaga Sakulploy, Kanokpan Pancharoen, Chariya Utenpitak, Caroline Fukuda, Thitima Cherdtrakulkiat, Tanyawarin Janthiraj, Anuwat Sriporn, Natee Prathummart, Patsaraporn Khongsom, Navakarn Navanuch, Rinda Wongbenchaporn, Chanya Peerapatdit, Pechpailin Khlaimanee, Patcharat Niyamakom, Narongritt Tippanont, Somsak Yafant, Tatchai Ruanpang, Siripak Pongthai, Kamolnetr Okanurak, Aronrag Meeyai, Danai Linjongrat, Phubet Panpet, Orawan Fungfoosri, Prisana Boonyawan, Theeranat Sangprasert, Natthawirojn Inthanin, Teppanan Sangiamjit, Somporn Saiwaew, Konlawat Pawong, Surang Janyam, Chamrong Phaengnongyang, Atachai Phunkron, Denchai Srikrongthong, Thanaphat Dokrak, Phathranis Meekrua, Saman Sumalu, Cawee Kanlose, Prasopsuk Thapwong, Kritsanapol Kaewboonta, Pornpichit Brutrat, Waris Watthanayeam, Apichat Udomjirasirichot, Somchai Sriplienchan, Midnight Poonkasetwattana, Silapakhon Kongsakul, Michael Badorrek, Andrey Tran, Ryan Figueiredo, Safir Soeparna, Wattana Keiangpa, Apiwit Tibamrung, Sunadda Samana, Hidayah Syahputra, Worapon Rattanawarawong, Patrick Sullivan, Rachel Valencia, Usha Sharma, Adeola Adeyeye, James Rooney, Pojjana Hunchangsith, Tanyaporn Wansom, Thomas Guadamuz, Annette Sohn

**Affiliations:** a Center for Public Health and Human Rights, Department of Epidemiology, Johns Hopkins Bloomberg School of Public Health, Baltimore, MD, USA; b Department of Health, Behavior & Society, Johns Hopkins Bloomberg School of Public Health, Baltimore, MD, USA; c Division of HIV Prevention, U.S. Centers for Disease Control and Prevention, Atlanta, GA, USA; d Division of HIV Prevention, Thailand Ministry of Public Health-U.S. Centers for Disease Control and Prevention Collaboration, Nonthaburi, Thailand; e Department of Population, Family & Reproductive Health, Johns Hopkins Bloomberg School of Public Health, Baltimore, MD, USA; f Department of International Health, Johns Hopkins Bloomberg School of Public Health, Baltimore, MD, USA; g Service Workers in Group Foundation (SWING), Bangkok and Pattaya, Thailand; h Rainbow Sky Association of Thailand (RSAT), Bangkok, Thailand; i Medical Affairs, Gilead Sciences, Foster City, CA, USA; j Department of Epidemiology, Emory University Rollins School of Public Health, Atlanta, GA, USA; k Division of AIDS, National Institute of Allergy and Infectious Diseases, National Institutes of Health, Bethesda, MD, USA; l Department of Epidemiology, Mahidol University, Nakhon Pathom, Thailand; m Asia Pacific Coalition on Male Sexual Health Foundation (APCOM), Bangkok, Thailand; n Duke Global Health Institute, Duke University, Durham, NC, USA

**Keywords:** HIV, Prevention, Pre-exposure prophylaxis, Men who have sex with men, Transgender persons, Sex work, Thailand

## Abstract

**Background:**

Daily oral pre-exposure prophylaxis (PrEP) is effective in preventing HIV infection, but no study has evaluated combination prevention interventions with PrEP for transgender women (TGW) and men who have sex with men (MSM) who sell sex.

**Methods:**

The Combination Prevention Effectiveness (COPE) study was a community-based, non-randomized implementation study in Bangkok and Pattaya, Thailand. Participants were HIV-negative MSM and TGW aged 18–26 years who reported exchanging sex with men in the prior 12 months and who met 2014 U.S. Public Health Service PrEP eligibility criteria. The intervention included quarterly HIV testing, semiannual testing for sexually transmitted infections, provision of condoms with lubricant, and the opportunity to initiate or end daily oral PrEP use at any time during study participation. Participants taking PrEP received monthly adherence counseling and short message service reminders. The primary outcome was HIV incidence rate ratio (IRR) on PrEP vs. not on PrEP. Secondary outcomes were PrEP initiation, PrEP use at 12 months, and PrEP adherence.

**Findings:**

From October 2017 to August 2019, 846 participants were enrolled: 531 (62.8%) immediately initiated PrEP; 104 (12.3%) subsequently initiated PrEP, and 211 (24.9%) never initiated PrEP. Among those initiating PrEP within 30 days of enrollment; 85.9% were on PrEP at the 12-months. When taking PrEP, participants reported adherent PrEP use at 94.2% of quarterly assessments. Ten HIV seroconversions occurred without PrEP use (incidence rate [IR] = 3.42 per 100 person-years [PY]; 95% CI = 1.64–6.30), while zero cases occurred with PrEP use (IR = 0.0 per 100PY; 95% CI = 0.0–0.62), with IRR = 0.0 (95% CI = 0.0–0.22; p < 0.001).

**Interpretation:**

Young Thai MSM and TGW who exchange sex can have high PrEP uptake, persistence and adherence, and low HIV incidence when offered in supportive community-based settings.

**Funding:**

U.S. National Institute of Allergy and Infectious Diseases; 10.13039/100000030Centers for Disease Control and Prevention.

**Research in context**

**Evidence before this study**

Daily oral pre-exposure prophylaxis (PrEP), when taken as directed, dramatically reduces risk of HIV infection, but real-world effectiveness is often limited by inadequate PrEP uptake, low rates of persistence, and inadequate adherence. A recent global systematic review of oral PrEP studies estimated that after six months of PrEP initiation, only 30% were still using PrEP with sufficient adherence to prevent HIV infection. The systematic review did not identify any longitudinal PrEP studies with MSM or TGW who sell or exchange sex, despite high HIV incidence in these key populations. While young people who sell sex are prioritized in many guidelines and recommendations, there have been remarkably few effectiveness studies in these populations.

We searched PubMed using the terms “HIV”, “MSM” or “TGW, “PrEP” or “oral pre-exposure prophylaxis”, and “sex worker” or “sex work” for articles written in English published through December 31, 2021. We also tracked PrEP studies enrolling MSM and/or TGW in Thailand on clinicaltrials.gov and the World Health Organization International Clinical Trials Registry Platform (WHOICTRP) using search terms “HIV”, “PrEP”, and “MSM” or “TGW” or “Men who have sex with men” or “Transgender women”.

**Added value of this study**

The COPE study used an open label non-randomized approach to assess the real-world effectiveness of a combination HIV prevention intervention with PrEP specifically developed with and for young MSM and TGW who sell or exchange sex. Novel study components included the implementation science design, use of social media influencers (SMI) to promote study engagement, and partnership with key population-led service providers. Study participants, aged 18–26 years at enrollment, had high PrEP uptake—including same-day PrEP initiation—high persistence over 12 months, and high adherence as measured by self-report, pill pickups, and a biological assay (for a correlation assessment). There were no HIV infections during PrEP use, whereas 10 infections occurred when participants were not using PrEP. This open label, implementation study offers important considerations for alternative means to evaluate the effectiveness of combination HIV prevention interventions as randomized controlled trials become more challenging to implement in a HIV prevention field with multiple efficacious options for prevention, including long-acting injectable cabotegravir, the dapivirine ring, and on-demand oral regimes.

**Implications of all the available evidence**

Key population-led combination HIV prevention interventions with PrEP for MSM and TGW who sell or exchange sex can dramatically reduce HIV incidence. Such interventions should be supported in Thailand and should be adapted, implemented, and evaluated in other cultural and social settings across the Asia-Pacific region and globally. The COPE study also shows that community engaged prevention research, including with key population delivered services provision, is feasible and can yield important insights into HIV prevention for those most at risk.

## Introduction

Thailand has a concentrated HIV epidemic among men who have sex with men (MSM), transgender women (TGW) who have sex with men,^1^ and sex workers of all genders.^2^, ^3^, ^4^ The highest HIV incidence densities in the country have been reported for young MSM and TGW, particularly the subset of these communities who sell or exchange sex in the large sex work sectors in Bangkok and Pattaya.^4^, ^5^, ^6^, ^7^ Estimates from a Bangkok-based cohort of young MSM and TGW (2006–2015) reported an incidence rate (IR) of 11.1 per 100 person-years (PY) (95% CI: 6.7–17.4) among those who sold or exchanged sex in the past four months.^4^

Despite clear vulnerability to HIV acquisition for the subset of young MSM and TGW who sell and exchange sex, prevention trials and implementation science have focused less on these groups than other MSM and TGW. The development and assessment of HIV prevention packages for young MSM and TGW who sell and exchange sex are needed in Thailand and elsewhere,^5^^,^^6^^,^^8^ and prevention packages need to be responsive to complex occupational contexts of sex work and social contexts of sex workers. Occupational contexts of sex work span the spectrum from the formal sex work industry reliant on physical venues such as bars, clubs, and saunas, to informal relationships of longer duration, which now exist against a backdrop of online spaces to meet potential clients.^9^^,^^10^ Young MSM and TGW may occasionally sell or trade sex but may not identify as a sex worker.^5^^,^^9^ Sex work and exchange sex in Thailand can have seasonal dynamics and be associated with mobility within and outside of the country.^9^ Taken together, these contexts create important considerations for HIV prevention, including diverse risk environments and dynamic behaviors and experiences.

HIV pre-exposure prophylaxis (PrEP) with daily oral tenofovir disoproxil fumarate/emtricitabine (TDF/FTC) PrEP, was approved for use in Thailand shortly after approval by the World Health Organization in 2013. However, use of daily oral PrEP remained low among Thai persons at high risk for HIV,^11^ and in 2017 there were only an estimated 1865 PrEP clients across Thailand.^12^ Globally, challenges with PrEP for HIV prevention, particularly among younger clients and those at higher risk of HIV acquisition from transactional sex, have been adherence and persistence.^13^, ^14^, ^15^ To evaluate whether a combination preventive intervention with the offer of free HIV PrEP could reduce HIV incidence among young MSM and TGW engaged in sex work, we developed and implemented an open label effectiveness study, the Combination Prevention Effectiveness (COPE) project. The following analyses assess the primary outcomes of PrEP uptake, PrEP adherence, 12-month PrEP adherence, and the reduction in HIV incidence associated with PrEP use among young MSM and TGW engaged in sex work.

## Methods

COPE was a combination prevention intervention for Thai MSM and TGW who sell sex that included an open-label offer to initiate and discontinue daily oral PrEP as desired. Given the established efficacy of daily oral PrEP, the open-label approach was preferred over a randomized clinical trial. There was no prospective assignment of study participants to treatment groups, and the study was determined by the sponsor not to be a clinical trial. Supplemental File S1 displays the study flow.

Development and implementation of the intervention was informed by qualitative research and by expertise of the community-based organizations that provide services to MSM and TGW sex workers^11^ and that would be recruiting participants, providing intervention services, and collecting data in the COPE study.^9^ Study sites included the Silom Community Clinic at the Hospital for Tropical Diseases or TropMed (Bangkok), Rainbow Sky Association of Thailand (Bangkok), and the Service Workers in Group Foundation (Bangkok and Pattaya). A separate manuscript describes the COPE study protocol and provides detailed description of sample size calculations, sampling methods, and other study components.^16^

### Study eligibility and recruitment

Eligibility criteria included: male sex at birth; 18–26 years of age; self-reported HIV-negative or unknown serostatus; sex with cisgender men for money, drugs, or other goods in the past 12 months; living in the greater Bangkok metropolitan area or Pattaya; and Thai citizenship.

During formative qualitative research, a number of participants assigned male sex at birth expressed identities on the transfeminine spectrum and expressed eagerness to participate in the proposed study.^9^ With strong support from Thai community partners, we expanded the eligibility criteria to included persons assigned male sex at birth with transfeminine gender identities. The terms MSM and TGW, as accepted in the scientific literature, were used in this study, although these are composite terms in Thailand that include approximately 15 unique identities embodying both sexual preferences and gender expression.^17^^,^^18^

**Fig. 1:**
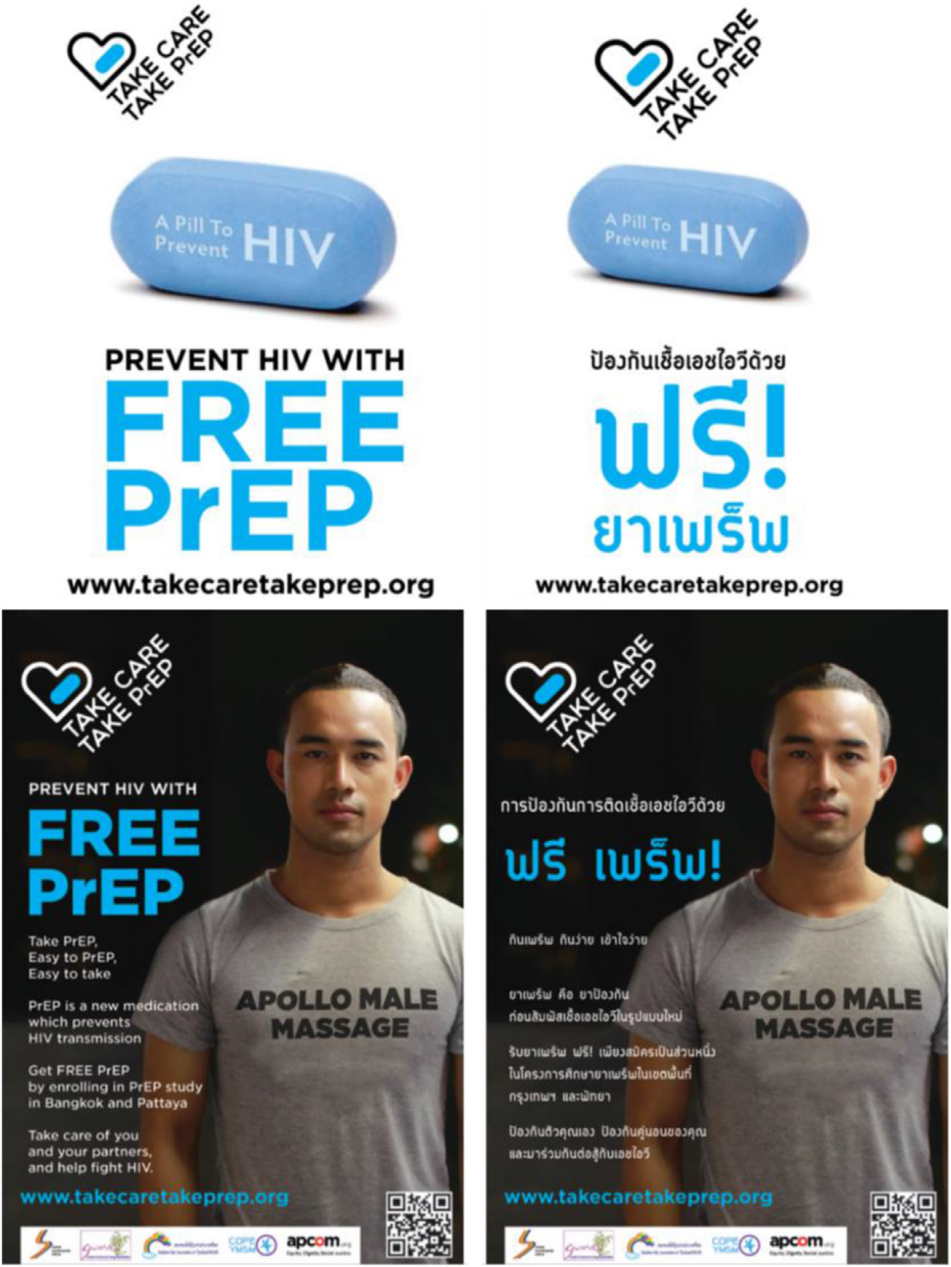
Static images used in the social media campaign for the Combination Prevention Effectiveness (COPE) study for young men who have sex with men and transgender women who exchange sex, Thailand (2018–2020). The social media campaign included static imagery and short promotional videos. The campaign was promoted on local social media platforms (Facebook, Twitter, and LINE), project partner websites and newsletters, and gay networking applications and forums (Hornet, Blued, Postjung, GThai, Planet Romeo, and Palm Plaza).

From October 2017 through August 2019, participants were recruited through outreach at physical sex work venues (e.g., bars, saunas, brothels, karaoke parlors), clinic-based referral, word-of-mouth, and a social media campaign disseminated through popular geosocial network applications for MSM and TGW (Fig. 1).^16^ Interested individuals were directed to call study staff to complete a pre-screening and schedule an in-person appointment at one of the study sites for further eligibility screening. Individuals who met eligibility criteria and provided informed consent were enrolled.

### Combination prevention intervention

#### Intervention components

The combination intervention included multiple individual-level components. All participants received HIV testing and counseling every three months. Screening for syphilis, rectal gonorrhea, and rectal chlamydia were conducted every six months, or as needed based on symptoms or participant request, with treatment for all identified sexually transmitted infections (STIs). Condoms and condom-compatible lubricant were offered at all study visits and were readily available at each study site. At the baseline assessment, all participants were offered the opportunity to start daily oral TDF/FTC PrEP (Truvada®, Gilead Sciences, Inc.), and participants could start, stop, and restart PrEP during study participation. Participants wishing to start PrEP needed to meet United States Centers for Disease Control and Prevention (CDC) guidance for PrEP eligibility: negative HIV test at the visit, including negative nucleic acid amplification test (NAAT) for those with acute symptoms; estimated creatinine clearance (eCrCl) ≥ 60 mL/min; and no major known allergy to TDF and/or FTC.^19^ PrEP counseling followed the US Public Health Service Preexposure Prophylaxis for the Prevention of HIV Infection in the United States—2014 Clinical Practice Guideline.^20^

For participants initiating PrEP, monthly pick-up visits included the following procedures: return of unused medication; assessment of signs and symptoms of acute HIV infection; assessment of side effects; adherence counseling informed by weekly short message service (SMS) survey responses and unused tablet count; collection of dried blood spots (DBS) for future adherence testing; and dispensation of one 30-tablet bottle of TDF/FTC PrEP. More than one bottle was provided if a participant would not be able to return in 30 days due to travel or other reason.

#### SMS adherence support

Participants were offered options for daily or weekly SMS adherence reminders, and participants were asked how many PrEP doses they had taken in the last seven days through a weekly SMS survey. FrontlineSMS (Occam Technologies, Washington, DC) was used for secure SMS-based communication and data collection. For adherence support and breakthrough HIV infection prevention, a participant was considered to have inadequate PrEP adherence if weekly SMS adherence measures indicated two consecutive weeks with zero to three doses taken per week or if more than 16 pills remained from the prior month during a medication pick-up. Participants with inadequate PrEP adherence could restart PrEP after re-screening for PrEP eligibility.

#### HIV testing

Participants were tested for HIV at the baseline and quarterly visits, when screened for PrEP initiation, and if reporting symptoms indicative of possible acute infection. HIV testing included rapid blood-based antibody testing (Determine, One Step, Wantai, or Bioline, depending on study site), supplemented by a reflex pooled NAAT for antibody-negative specimens. All reactive results were confirmed by additional rapid test and/or NAAT (following contemporaneous Thai national HIV testing guidance), and participants with confirmed HIV infection were linked to HIV care and treatment.

#### Sexually transmitted infection testing

Rectal gonorrhea or chlamydia infection was diagnosed by NAAT-positive rectal swab that was either provider-collected or self-collected, depending on participant preference. Venipuncture blood specimens were tested for antibody to *Treponema pallidum* (*T. pallidum*) by rapid plasma reagin (RPR) reactivity and titer and *T.* pallidum antibody rapid test. Persons who also tested *T. pallidum* antibody positive and with RPR ≥1:8 were considered seropositive for syphilis. Participants with positive STI results were provided oral or injection treatment following local clinical standards.

#### Study exit

Study exit categories included study completion, HIV diagnosis, lost to follow-up, and withdrawn. Participants were considered to have completed the study if they completed the last expected protocol-defined study visit or if their study participation was stopped due to intervention phase close-out at their study site. Laboratory-confirmed HIV infection was considered incident at the date of diagnosis. Participants with no study visits for more than 300 days were considered lost to follow-up at their last completed study visit. Participants were withdrawn from the study if they reported experiencing serious adverse events, permanently relocated outside of Bangkok and Pattaya, or withdrew consent for study participation, or if continued participation could reasonably result in physical or social harm.

### Measures

The primary outcome was the HIV incidence rate ratio (IRR) on PrEP vs. not on PrEP. Secondary outcomes included PrEP initiation, PrEP adherence, and persistent PrEP use at 12 months, and IRRs for rectal gonorrhea, chlamydia, and syphilis. Operational definitions are defined in Table 1.

**Table 1:** Outcome measures, sub-components, and their definitions in the Combination Prevention Effectiveness (COPE) study for young men who have sex with men and transgender women who exchange sex, Thailand (2018–2020).

Outcome measure and sub-components	Operational definition
**HIV incidence**	**HIV infections not on PrEP per 100 PY not on PrEP among all study participants; HIV infections on PrEP per 100 PY on PrEP among all study participants**
HIV infection	Positive HIV Ab and positive NAAT
PrEP status at HIV infection	PrEP status at midpoint between last negative and first positive HIV tests
Time on PrEP	Days between starting PrEP and stopping PrEP or study exit
Time not on PrEP	Days between study entry and starting PrEP or study exit; days between stopping PrEP and starting PrEP or study exit
PrEP initiation	Date first received study PrEP after enrollment or after having stopped PrEP use for more than 14 days
Stopping PrEP	Date study PrEP would have run out with daily use and did not receive additional study PrEP within 14 days
**Same-day PrEP initiation**	**Received study PrEP on day of study enrollment**
**PrEP persistence**	**Among participants initiating PrEP within 30 days of enrollment, on PrEP at the 12-month assessment regardless of any time off PrEP in the interim**
**Adherence**	**Self-report of taking 4 or more PrEP doses in the last 7 days if not using exogenous feminizing hormones; self-report of taking 7 doses in the last 7 days if using exogenous feminizing hormones**
Exogenous feminizing hormone use	Self-report of using exogenous feminizing hormones since the prior study visit
**[Chlamydia/gonorrhea/syphilis] incidence**	**[Chlamydia/gonorrhea/syphilis] infections per 100 PY not on PrEP among all study participants; [chlamydia/gonorrhea/syphilis] infections per 100 PY on PrEP among all study participants**
Rectal chlamydia infection	*Positive NAAT result from rectal swab*
Rectal gonorrhea infection	*Positive NAAT result from rectal swab*
Syphilis infection	*Treponema pallidum* detection by rapid plasma reagin (RPR) ≥ 1:8 and *T. pallidum* antibody positive
PrEP status at [chlamydia/gonorrhea/syphilis] infection	PrEP status at midpoint between positive [chlamydia/gonorrhea/syphilis] test and prior [chlamydia/gonorrhea/syphilis] test

PrEP, pre-exposure prophylaxis for HIV; PY, person-years; Ab, antibody test; NAAT, nucleic acid amplification test.

#### PrEP use status

PrEP use was determined based on PrEP dispensation records. Participants were considered to have initiated PrEP when they first received study PrEP pills or when they received study PrEP pills after a period of at least two weeks of no PrEP use. Participants were considered to have stopped PrEP use when they would have exhausted their existing study PrEP supply with daily use and they subsequently did not receive additional study PrEP within 14 days.

PrEP use status for newly diagnosed HIV infections was *a priori* defined as PrEP use at the midpoint between the last negative HIV test and first positive HIV test.^16^ We complemented the *a priori* classification of PrEP use with a sensitivity analysis using imputation methods. PrEP use status for a given STI test was defined by PrEP use at the midpoint between the prior and the given STI test.

#### Self-reported adherence

Participants were asked about PrEP use in the last seven days during each quarterly assessment and through weekly SMS surveys. PrEP adherence was defined as the participant self-reporting taking PrEP pills on four or more days out of the last seven days if not using exogenous feminizing hormones or taking PrEP on seven of the last seven days if using exogenous feminizing hormones. These weekly use thresholds have previously been correlated with protective blood levels of tenofovir diphosphate (TFV-DP).^21^ Adherence based on quarterly assessments was used as the primary measure of adherence, but we also report adherence based on SMS survey response. Self-reported PrEP use and adherence were validated with a sub-sample analysis of TFV-DP levels in DBS from 120 quarterly assessments among 30 participants. TFV-DP concentrations above 700fmol/3 mm punch were considered indicative of good adherence^22^ (Supplemental File S3).

### Analyses

Descriptive statistics are provided for the following: immediate, delayed, and no PrEP initiation; person-time on and off of PrEP; persistence on PrEP at the 12-month assessment among participants initiating PrEP within 30 days of study enrollment; and PrEP adherence. For evaluating incidence of each STI, person-time extended to the last STI test, and person-time was categorized as on PrEP or off PrEP.

For evaluating HIV incidence, person-time extended to the date of first positive HIV test for participants with seroconversion or last negative HIV test for participants without seroconversion, with person-time categorized into on PrEP or off PrEP. In the *a priori* analysis, the number of incident HIV infections while on PrEP and number of incident HIV infections while off PrEP were based on PrEP use at the midpoint between the last negative HIV test and first positive HIV test. For both HIV and STIs, exact 95% confidence intervals (CIs) for IRRs and incidence rates were calculated using probabilities from the binomial distribution, and p-values for HIV and STI IRRs are two-sided exact p-values based on the binomial distribution.^23^ Due to the limited number of HIV infections, covariate-adjusted parametric models were not tenable. We conducted a sensitivity analysis of HIV incidence using multiple imputations to randomly select a date of HIV infection between the last negative HIV test and first positive HIV test (Supplemental File S4). All analyses were conducted with Stata 17.

### Ethical considerations

The protocol was reviewed and approved by the Thailand Ministry of Public Health Ethical Review Committee for Research in Human Subjects, the CDC institutional review board (IRB), the Johns Hopkins Bloomberg School of Public Health IRB, and the Prevention Sciences Review Committee of the Division of AIDS, U.S. National Institute of Allergy and Infectious Diseases/National Institutes of Health. Participants were compensated for study participation. For participants who initiated PrEP, the total possible compensation for one year of follow-up was 14,360 Thai Baht (THB) ($479 USD). For those who never initiated PrEP, the total possible compensation for one year was 8360 THB ($279 USD). A detailed compensation schedule is provided in Supplemental File S2.

### Role of funding source

The NIH had no role in the study design; collection, analysis, or interpretation of data; writing of this report; or decision to submit for publication. CDC study staff contributed to the study design; data collection; writing of this report; and the decision to submit for publication.

## Results

**Fig. 2:**
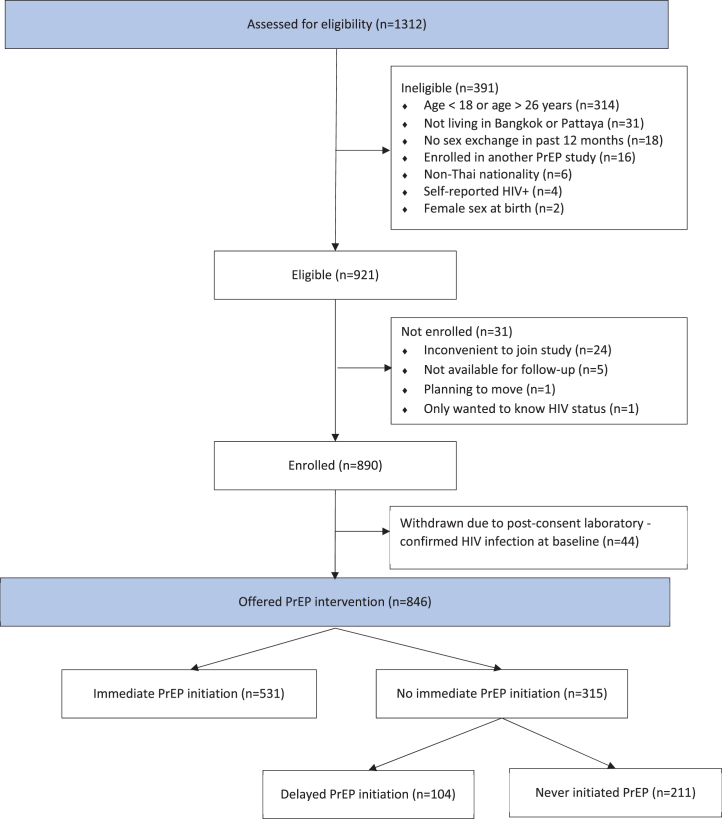
Screening and enrollment in the COPE study for young men who have sex with men and transgender women who exchange sex, Thailand (2018–2020). PrEP, pre-exposure prophylaxis. Immediate PrEP initiation defined as starting PrEP on day of study enrollment. Delayed PrEP initiation defined as starting PrEP after study entry.

A total of 1312 individuals were screened for study eligibility, with 921 meeting eligibility criteria, and 890 providing informed consent and enrolling in the study (Fig. 2, Supplemental File S5). Forty-four participants (4.9%) were found to be HIV seropositive at the baseline assessment, excluded from further study participation, and referred for treatment; 846 participants contributed person-time on study.

Most of the 846 participants were enrolled at the Silom Community Clinic at TropMed (56.7%), with the remainder enrolled at community sites (Table 2). Slight majorities of participants had at least a university education (54.1%) or were currently in school (53.2%), and most respondents (89.4%) reported having sufficient money to meet basic needs at least most of the time. Most participants (89.6%) self-identified as men, and 8.6% self-identified as a woman or transgender woman. At the baseline assessment, 85.4% of participants had ever heard of PrEP, 81.3% knew where they could get PrEP, 76.4% were willing to take PrEP, and 17.9% reported prior PrEP use.

**Table 2:** Baseline characteristics and PrEP initiation among participants in the COPE study for young men who have sex with men and transgender women who exchange sex, Thailand (2018–2020) (n = 846).

	All participants	Initiation of PrEP use during study participation			
	N	Immediate n (%)	Delayed n (%)	None n (%)	p-value
	846	531 (62.8%)	104 (12.3%)	211 (24.9%)	
**Study site**					<0.001
Silom Community Clinic at Tropmed	480	319 (66.5%)	70 (14.6%)	91 (19.0%)	
Service Workers in Group Foundation Bangkok	133	86 (64.7%)	17 (12.8%)	30 (22.6%)	
Rainbow Sky Association of Thailand	207	123 (59.4%)	15 (7.2%)	69 (33.3%)	
Service Workers in Group Foundation Pattaya	26	3 (11.5%)	2 (7.7%)	21 (80.8%)	
**Sociodemographic**					
Years of age					0.18
18-20	189	117 (61.9%)	20 (10.6%)	52 (27.5%)	
21-23	359	214 (59.6%)	47 (13.1%)	98 (27.3%)	
24-26	297	200 (67.3%)	37 (12.5%)	60 (20.2%)	
Completed University education					0.018
No	388	224 (57.7%)	54 (13.9%)	110 (28.4%)	
Yes	457	307 (67.2%)	50 (10.9%)	100 (21.9%)	
Currently at school					0.42
No	396	249 (62.9%)	54 (13.6%)	93 (23.5%)	
Yes	450	282 (62.7%)	50 (11.1%)	118 (26.2%)	
Income from work, including sex work					0.40
No	244	147 (60.2%)	29 (11.9%)	68 (27.9%)	
Yes	581	371 (63.9%)	74 (12.7%)	136 (23.4%)	
Sufficient income to meet basic needs					0.71
No	88	58 (65.9%)	11 (12.5%)	19 (21.6%)	
Yes	744	462 (62.1%)	92 (12.4%)	190 (25.5%)	
Currently in a relationship					0.52
No	529	338 (63.9%)	60 (11.3%)	131 (24.8%)	
Yes	316	193 (61.1%)	44 (13.9%)	79 (25.0%)	
Gender identity^a^					0.046
Man, gay, or bisexual	758	489 (64.5%)	89 (11.7%)	180 (23.7%)	
Woman or transgender woman	73	35 (47.9%)	12 (16.4%)	26 (35.6%)	
Other or don't know	15	7 (46.7%)	3 (20.0%)	5 (33.3%)	
**Sexual behavior**					
Last time had anal sex with man					0.008
Within 30 days	518	340 (65.6%)	68 (13.1%)	110 (21.2%)	
More than 30 days	149	85 (57.0%)	12 (8.1%)	52 (34.9%)	
Don't know or refused to answer	179	106 (59.2%)	24 (13.4%)	49 (27.4%)	
Used condom during last anal sex					<0.001
No	214	104 (48.6%)	50 (23.4%)	60 (28.0%)	
Yes	615	422 (68.6%)	52 (8.5%)	141 (22.9%)	
Don't know	17	5 (29.4%)	2 (11.8%)	10 (58.8%)	
Used alcohol or drugs during anal sex in last 7 days					0.65
No	512	321 (62.7%)	58 (11.3%)	133 (26.0%)	
Yes	91	61 (67.0%)	11 (12.1%)	19 (20.9%)	
Don't know or refused to answer	243	149 (61.3%)	35 (14.4%)	59 (24.3%)	
**Clinical testing**					
Tested for HIV in last 6 months					<0.001
No	272	142 (52.2%)	42 (15.4%)	88 (32.4%)	
Yes	574	389 (67.8%)	62 (10.8%)	123 (21.4%)	
Chlamydia infection					0.026
No	722	442 (61.2%)	88 (12.2%)	192 (26.6%)	
Yes	124	89 (71.8%)	16 (12.9%)	19 (15.3%)	
Gonorrhea infection					0.060
No	803	498 (62.0%)	98 (12.2%)	207 (25.8%)	
Yes	42	32 (76.2%)	6 (14.3%)	4 (9.5%)	
Syphilis infection					0.25
No	799	497 (62.2%)	98 (12.3%)	204 (25.5%)	
Yes	47	34 (72.3%)	6 (12.8%)	7 (14.9%)	
**Pre-exposure prophylaxis (PrEP)**					
Ever heard of PrEP before					0.11
No	123	67 (54.5%)	19 (15.4%)	37 (30.1%)	
Yes	722	464 (64.3%)	85 (11.8%)	173 (24.0%)	
Ever taken PrEP before					<0.001
No	694	412 (59.4%)	91 (13.1%)	191 (27.5%)	
Yes	151	119 (78.8%)	13 (8.6%)	19 (12.6%)	
Know where to get PrEP					0.21
No	158	90 (57.0%)	21 (13.3%)	47 (29.7%)	
Yes	687	441 (64.2%)	83 (12.1%)	163 (23.7%)	
Know others who use PrEP					0.001
No	461	266 (57.7%)	59 (12.8%)	136 (29.5%)	
Yes	384	265 (69.0%)	45 (11.7%)	74 (19.3%)	
Willing to take PrEP every day					<0.001
No	199	50 (25.1%)	30 (15.1%)	119 (59.8%)	
Yes	646	481 (74.5%)	74 (11.5%)	91 (14.1%)	

p-values based on Pearson's chi-squared test comparing all three PrEP initiation categories.

Immediate PrEP initiation defined as starting PrEP on day of study enrollment.

Delayed PrEP initiation defined as starting PrEP after day of study entry.

a Gender identity classification reported here reflects multiple identities in Thailand that embody both sexual and gender preferences and expression; see text.

### PrEP use

During participation, 531 (62.8%) initiated PrEP use at baseline, 104 (12.3%) initiated PrEP use after the baseline assessment, and 211 (24.9%) never initiated PrEP. Several baseline factors were associated with immediate and delayed PrEP initiation, including recency of anal intercourse, condom use at last intercourse, laboratory-confirmed chlamydia infection, past PrEP use and PrEP awareness (Table 2).

### Persistence of PrEP use

Among participants initiating PrEP within 30 days of the baseline assessment (n = 574), 91.1% were using PrEP at the three-month assessment, 89.0% at the six-month assessment, 84.8% at the nine-month assessment, and 85.9% at the 12-month assessment.

### PrEP adherence

Number of days of PrEP use within the last seven days was reported at 2367 quarterly follow-up assessments. Of these participant self-reports, 2230 (94.2%) were indicative of good adherence to daily oral PrEP. During study time included in the outcome analyses, 37,370 weekly SMS surveys were completed by 766 participants (90.5%), covering 716.2 PY (80.9%) of the 885.1 PY in the outcome analyses. Participants were asked about PrEP use during the last seven days in 27,590 (73.8%) of these completed SMS surveys, with 96.2% of responses indicative of good adherence.

In a subsample of DBS from participants with self-reported adherent use, 53 of 66 (80.3%) had sufficient TFV-DP levels (>700 fmol/3 mm punch). The overall kappa statistic indicated very good agreement between PrEP adherence based on self-reported and PrEP adherence based on TFV-DP levels (81.7%; *χ*^2^ = 82.6, p < 0.001).^24^ See Supplemental File S3 for additional DBS results.

### HIV incidence

**Fig. 3:**
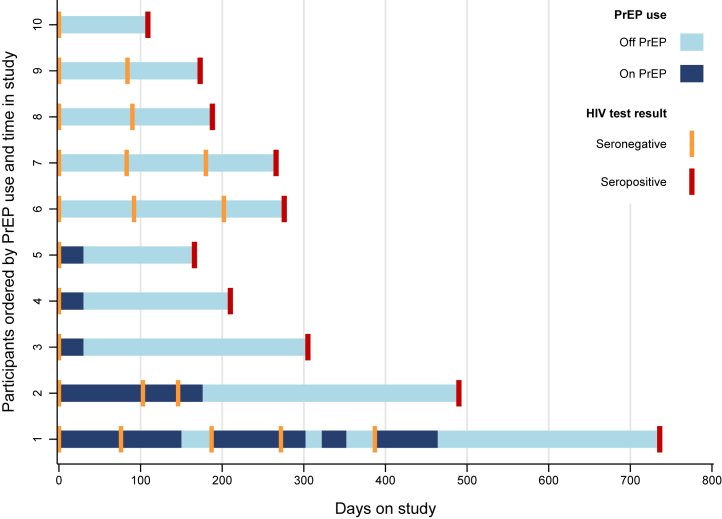
Patterns of PrEP use and HIV testing among participants with HIV seroconversion in the COPE study for young men who have sex with men and transgender women who exchange sex, Thailand (2018–2020). PrEP, pre-exposure prophylaxis.

Overall, there were 885.1 PY in the HIV incidence analyses, including 593.1 PY on PrEP and 292.0 PY not on PrEP. Ten HIV seroconversions were observed among participants over the course of the study, all of whom identified as MSM at the baseline assessment. Five participants with HIV seroconversion never received PrEP through the COPE study and five were dispensed PrEP at the baseline assessment and at the time of their last negative HIV test (Fig. 3). Between the last negative HIV and first positive HIV test, four participants received 30 PrEP pills and one participant received 60 PrEP pills over two visits. All five HIV-seroconverting participants who never received PrEP consistently completed quarterly assessments, with approximately three months (74–109 days) between their last negative HIV test and their first HIV positive test. All five HIV-seroconverting participants receiving PrEP had long durations between their last negative and first positive HIV test (166, 210, 305, 344, and 349 days). The proportion of these days covered by PrEP use ranged from 0.087 to 0.181 (Fig. 3).

Using the *a priori* rule that PrEP use at seroconversion would be based on PrEP use at the midpoint between the last negative HIV test and first positive HIV test, all ten incident HIV infections would be assumed to have occurred while not using PrEP. This corresponds to an incidence rate of 0.0 per 100 PY on PrEP (95% CI = 0.0–0.62), 3.42 per 100 PY not on PrEP (95% CI = 1.64–6.30), and an IRR of 0.0 (95% CI = 0.0–0.22; p < 0.001) (Table 3). Sensitivity analysis based on imputed datasets provided an IRR of 0.041 (95% CI = 0.006–0.366; p < 0.001) (Supplemental File S4).

**Table 3:** Chlamydia, gonorrhea, syphilis, and HIV incidence during time on PrEP and time not on PrEP among participants in the COPE study for young men who have sex with men and transgender women who exchange sex, Thailand (2018–2020) (n = 844).

Infection detected	Incident cases	Incidence per 100 PY	(95% CI)	Incidence rate ratio	(95% CI)	p-value
*Chlamydia trachomatis*						
On PrEP	192	32.3	(27.9, 37.2)	1.64	(1.18, 2.33)	0.002
Not on PrEP	44	19.7	(14.3, 26.4)			
*Neisseria gonorrhoeae*						
On PrEP	79	13.3	(10.5, 16.6)	2.48	(1.34, 5.00)	0.002
Not on PrEP	12	5.4	(2.8, 9.4)			
*Treponema pallidum*						
On PrEP	76	12.8	(10.1, 16.0)	1.35	(0.82, 2.30)	0.269
Not on PrEP	21	9.5	(5.9, 14.5)			
HIV						
On PrEP	0	0.00	(0.00, 0.62)	0.00	(0.00, 0.22)	<0.001
Not on PrEP	10	3.42	(1.64, 6.30)			

PrEP, HIV pre-exposure prophylaxis; PY, person-years; CI, confidence interval; 95% confidence intervals and p-values from exact tests.

What might have been the HIV incidence if no participants had been offered PrEP? If the observed incident rate of 2.98 (95% CI = 1.43–5.48) per 100 PY without PrEP use is applied to time on PrEP, then an expected 18 (95% CI = 9–33) additional HIV infections would have occurred, for an expected total number of infections of 28 (95% CI = 19–43).

### STI incidence

STIs were common at baseline (Table 2), with 14.7% positive for rectal chlamydia, 5.0% positive for rectal gonorrhea, and 9.8% positive for syphilis, with generally higher prevalence at baseline among participants who initiated PrEP during study participation. During follow-up (Table 3), person-time on PrEP vs. not on PrEP was significantly associated with higher incidence of chlamydia (IRR = 1.64; 95% CI = 1.18–2.33) and gonorrhea (IRR = 2.48; 95% CI = 1.34–5.00), but not syphilis.

## Discussion

In this multilevel HIV prevention intervention with open-label PrEP, study participants—young Thai MSM and TGW who exchange or sell sex—had high rates of PrEP initiation (75.1%) and near universal weekly adherence and PrEP persistence among those initiating PrEP within 30 days of baseline. Most importantly, no HIV infections occurred during nearly 600 person-years of PrEP use in this otherwise high HIV incidence population. Despite 64% of total participant study-time covered by PrEP use, the ten seroincident HIV infections all occurred during time without PrEP use, corresponding to an IRR for person-time on PrEP vs. not on PrEP of 0.0 (95% CI = 0.0–0.25; p < 0.001). Sensitivity analyses accounting for uncertainty in PrEP use at time of seroconversion also yielded a very small IRR of 0.041 (95% CI = 0.006–0.366), corresponding to an estimated 95.9% lower rate of HIV infection (95% CI = 63.4%–99.4%). Based on the observed incidence rate without PrEP use, we estimate that there would have been 18 (95% CI = 9–33) additional HIV infections among study participants had no one been offered PrEP.

The HIV incidence among all study participants is also far lower than in a comparable external cohort, the Observational Clinical Cohort from the Silom Community Clinic.^25^ An unpublished subset analysis of MSM aged 18-26 years-old attending the SCC clinic and having at least two HIV tests from October 2017 through December 2018—a time period and a location overlapping with the COPE study activities—showed that 18 HIV infections occurred over 232.7 PY, corresponding to an incidence rate of 7.7 per 100 PY (95% CI = 4.6–12.2) (personal communication, Eileen F. Dunne, CDC). The same incidence rate in the COPE cohort would have resulted in 72 infections (95% CI = 43–114). However, the extent to which the hypothetical 60 additional HIV infections would have been due to intervention effects in the COPE study or to differences in study populations remains an open question.

The marked reduction in HIV incidence with PrEP use is consistent with the high levels of adherence achieved by study participants. Based on self-report, participants had adherent PrEP use during 96.4% of the weeks while on PrEP, and the validity of self-reports is largely supported by the DBS sub-sample analysis. When sufficient PrEP use was self-reported, 80.3% of samples had protective levels of TFV-DP levels, and even when insufficient PrEP use was reported, 42.9% of samples had protective levels of TFV-DP levels.

The outcomes in this study contrast with a recent meta-analysis of oral PrEP that estimated 31.5% PrEP discontinuation and 35.8% inadequate PrEP adherence globally among gay, bisexual, and other MSM and TGW on daily PrEP.^26^ High PrEP adherence has been observed in the Princess PrEP, a Thai key-populations-led intervention providing HIV testing and offering PrEP. Among MSM and TGW, adequate self-reported adherence was over 95%.^11^ However, persistence of PrEP use has been challenging: 24.6% for MSM and 18.5% for TGW after three months of PrEP initiation.^27^

Intervention components in the COPE study that may have supported high PrEP persistence and adherence include adherence counseling, pill reminders, and weekly SMS surveys. SMS reminders have been demonstrated to improve adherence to antiretroviral therapy and other public health interventions,^28^ although the emerging evidence base on SMS for PrEP adherence is mixed.^29^, ^30^, ^31^, ^32^ The SMS questions on PrEP use allowed a clinical protocol with a quick response to inadequate adherence. Although the weekly SMS questions on condomless anal intercourse and exchanging sex were intended as research measures of HIV risk, responding to these questions may have provided participants with feedback about their HIV risk which may have motivated PrEP use and adherence as a means of reducing risk. A recent randomized controlled trial evaluated a phone app to support PrEP adherence for young MSM and TGW in Thailand,^33^ The phone app was based on the information–motivation–behavioral model of behavior change and included weekly HIV risk assessment, daily reminders, or app use rewards. While assignment to the phone app condition was not associated with significant improvements in PrEP adherence at 6 months in this small sample study (n = 200), participants reported the risk assessment to be the most useful of the three app components.

The high interest in PrEP among study participants may also have contributed to the marked reduction in HIV incidence with PrEP use. The unmet demand for PrEP in this population of sex workers is indicated by the 75% of participants who initiated PrEP during study participation. The social media campaign and other study materials presented the COPE study as a PrEP-based study and sought to normalize PrEP use and stress the importance of adherence among MSM and TGW who sell or exchange sex, and this approach may have contributed to a study sample with a high interest in PrEP. Furthermore, the fact that 63% of participants initiated PrEP on the same day as study enrollment evinces the feasibility, acceptability and promise of same-day PrEP for achieving high PrEP uptake. Ensuring same-day PrEP access may be essential for PrEP-based HIV prevention for key populations in Thailand and elsewhere.

In 2021, key population led health services (KPLHS) provided PrEP for approximately two-thirds of the 13,769 PrEP clients in Thailand,^12^ and PrEP use stigma appears lower among KPLHS PrEP clients than hospital PrEP clients.^34^ Expanding combination HIV prevention interventions with PrEP through these organizations will require increasing the program space in which these providers operate and sustainability will require additional domestic funding. In addition to demonstrating the effectiveness and feasibility of this approach, building coalitions with multiple domestic and international stakeholders may support successful integration of key population based PrEP interventions into the national healthcare system.^35^

This study was an open-label effectiveness study in which treatment status was determined by participants who could start and stop PrEP use over the course of study participation. This design is a powerful approach to evaluating the uptake and effect of PrEP in preventing HIV acquisition among individuals with high risk of exposure in a context when random assignment to PrEP vs. placebo is no longer an ethically valid approach. However, this design does have limitations. HIV incidence during time off PrEP may not be an unbiased counterfactual for HIV incidence during time on PrEP, and therefore the incidence rate ratio for time on PrEP vs. time off PrEP may be a biased treatment effect estimate of PrEP use. The treatment effect may have been overestimated if, for example, PrEP uptake was higher among participants with lower risk for HIV. Conversely, the treatment effect may have been underestimated if, for example, uptake of PrEP was driven by treatment-by-indication, where participants were more likely to initiate PrEP use when they were at higher risk of infection, and the incidence rate without PrEP use would have been higher than observed for time off PrEP. The significantly higher incidence rates for chlamydia and gonorrhea for person-time on PrEP vs. not on PrEP observed in this study suggest that PrEP use was associated with periods of higher rather than lower risk of sexually transmitted HIV infection.

The estimated treatment effect is also predicated on a comprehensive, multilevel intervention package. Future studies should examine the importance of different components for reducing incidence, including social media and community-based campaigns, participant incentives, SMS risk and adherence-related mini-surveys, and prompt intervention when adherence falters.

There are also important considerations of external validity for other groups, including MSM and TGW who sell or exchange sex and are less interested in PrEP, members of other marginalized populations in Thailand, and MSM and TGW who sell or exchange sex in other parts of Thailand or in other countries. First, MSM and TGW who sell or exchange sex with lower PrEP awareness and interest may have lower PrEP uptake and adherence. Campaigns promoting and normalizing PrEP use may play an important role in increasing PrEP effectiveness for some individuals, and PrEP may simply be an unacceptable or otherwise inappropriate prevention strategy for other individuals. Second, minors involved in exploitative sex work are at high risk of HIV infection,^36^ and we were not able to enroll participants under 18 based on guidance from the Thai Ministry of Health Ethics Commission. Likewise, non-Thai national immigrants were excluded from participation due to their lack of eligibility for the Thai national health insurance program, but these same types of exclusions are often associated with higher risk of HIV infection among people who sell or exchange sex and other key populations.^37^ Third, MSM and TGW who sell or exchange sex in Bangkok and Pattaya Thailand may be more socially and politically cohesive and less marginalized than in other settings. Whether individual and group empowerment and dignity are important determinants to the success of PrEP uptake, adherence, and persistence in other settings should be considered.

As additional biomedical options for HIV prevention become available, randomized controlled trials and placebo arms will become increasingly challenging for the HIV prevention field. Open label observational effectiveness studies, such as the COPE study, offer important alternatives through which to assess relative effectiveness of interventions, including combination preventive packages with and without PrEP. The COPE study demonstrates that young Thai MSM and TGW who sell or exchange sex can and will successfully choose and adhere to combination regimens if these options are offered in safe, supportive, community-based settings. Since young MSM and transgender women are at very high risk for HIV acquisition across the Asia-Pacific region, these findings may have regional import and this intervention should be implemented and evaluated in other cultural and social settings.

## Contributors

Authors contributing to the writing and editing of the manuscript include BWW, ALW, CB, TC, SDB, SHHM, SO, JPN, NQ, JFR, HIS, JMT, PS, ACH, and TH. A full matrix of author contributions by study activities is provided in Supplemental File S6.

## Data sharing statement

Data sharing is limited by agreement with the Thailand Ministry of Public Health Ethics Review Committee. Any data requests can be sent to Andrea Wirtz (awirtz1@jhu.edu) for further deliberation by the principal investigators of the study.

## Declaration of interests

The study was supported by an R01 from NIAID, NIH (1R01AI118505-01A1) and the CDC Division of HIV Prevention. The study drug, Truvada®, was manufactured and donated by Gilead Sciences, Inc. Gilead Sciences, Inc. had no role in the design of the study nor in the interpretation of study results. The findings and conclusions presented in this paper are those of the authors and do not necessarily represent the views of the NIH, the US CDC, or 10.13039/100007197U.S. Public Health Service. Patrick Sullivan reports support from NIH, CDC, Merck, Gilead Sciences, ViiV Healthcare, Molecular Testing Labs, and Elsevier. James F. Rooney reports support from Gilead Sciences.

Rest of the authors declare no competing interests.

## Appendix A Supplementary data

Supplemental Files S1–S6

Translated abstract in Thai

Beyrer_COPE Study_Study Group for Indexing

## References

[1] van Griensven F., Thienkrua W., McNicholl J. (2013). Evidence of an explosive epidemic of HIV infection in a cohort of men who have sex with men in Thailand. AIDS.

[2] Muccini C., Crowell T.A., Pinyakorn S. (2021). Brief report: syphilis incidence and effect on viral load, CD4, and CD4/CD8 ratio in a Thai cohort of predominantly men who have sex with men living with HIV. J Acquir Immune Defic Syndr.

[3] Wansom T., Pinyakorn S., Kolsteeg C.J. (2020). Brief report: group sex and methamphetamine use fuel an explosive epidemic of hepatitis C among HIV-infected men who have sex with men in Bangkok, Thailand. J Acquir Immune Defic Syndr.

[4] Dunne E.F., Pattanasin S., Chemnasiri T. (2019). Selling and buying sex in the city: men who have sex with men in the Bangkok men who have sex with men Cohort Study. Int J STD AIDS.

[5] Baral S.D., Friedman M.R., Geibel S. (2014). Male sex workers: practices, contexts, and vulnerabilities for HIV acquisition and transmission. Lancet.

[6] Beyrer C., Crago A.L., Bekker L.G. (2014). An action agenda for HIV and sex workers. Lancet.

[7] Poteat T., Wirtz A.L., Radix A. (2015). HIV risk and preventive interventions in transgender women sex workers. Lancet (London, England).

[8] Poteat T., Scheim A., Xavier J., Reisner S., Baral S. (2016). Global epidemiology of HIV infection and related syndemics affecting transgender people. J Acquir Immune Defic Syndr.

[9] Truong J.M., Chemnasiri T., Wirtz A.L. (2022). Diverse contexts and social factors among young cisgender men and transgender women who sell or trade sex in Bangkok and Pattaya, Thailand: formative research for a PrEP program implementation study. AIDS Care.

[10] Weir B., Dun C., Wirtz A. (2022). Transactional sex, HIV and health among young cisgender men and transgender women who have sex with men in Thailand. Ann Epidemiol.

[11] Phanuphak N., Sungsing T., Jantarapakde J. (2018). Princess PrEP program: the first key population-led model to deliver pre-exposure prophylaxis to key populations by key populations in Thailand. Sex Health.

[12] Rayanakorn A., Chautrakarn S., Intawong K. (2022). A comparison of attitudes and knowledge of pre-exposure prophylaxis (PrEP) between hospital and key population led health service providers: lessons for Thailand's Universal health coverage implementation. PLoS One.

[13] Tangmunkongvorakul A., Chariyalertsak S., Amico K.R. (2013). Facilitators and barriers to medication adherence in an HIV prevention study among men who have sex with men in the iPrEx study in Chiang Mai, Thailand. AIDS Care.

[14] Farley J.E., Dangerfield D.T., LaRicci J. (2021). Community engagement and linkage to care efforts by peer community-health workers to increase PrEP uptake among sexual minority men. Public Health Nurs.

[15] Fields E.L., Hussen S.A., Malebranche D.J. (2020). Mind the gap: HIV prevention among young black men who have sex with men. Curr HIV/AIDS Rep.

[16] Wirtz A.L., Weir B.W., Mon S.H.H. (2020). Testing the effectiveness and cost-effectiveness of a combination HIV prevention intervention among young cisgender men who have sex with men and transgender women who sell or exchange sex in Thailand: protocol for the combination prevention effectiveness study. JMIR Res Protoc.

[17] Jackson P.A. (2000). An explosion of Thai identities: global queering and re-imagining queer theory. Cult Health Sex.

[18] Ocha W. (2012). Transsexual emergence: gender variant identities in Thailand. Cult Health Sex.

[19] CDC (2017).

[20] CDC (2014).

[21] Grant R.M., Anderson P.L., McMahan V. (2014). Uptake of pre-exposure prophylaxis, sexual practices, and HIV incidence in men and transgender women who have sex with men: a cohort study. Lancet Infect Dis.

[22] Deutsch M.B., Glidden D.V., Sevelius J. (2015). HIV pre-exposure prophylaxis in transgender women: a subgroup analysis of the iPrEx trial. Lancet HIV.

[23] Kleinbaum D.G., Kupper L.L., Morgenstern H. (1982). Epidemiologic research: principles and quantitative methods. Biometrics.

[24] Byrt T. (1996). How good is that agreement?. Epidemiology.

[25] Wimonsate W., Pattanasin S., Ungsedhapand C. (2018). Repeat HIV testing among HIV-uninfected men who have sex with men attending Silom Community Clinic, Bangkok, 2011 − 2014. Int J STD AIDS.

[26] Zhang J., Li C., Xu J. (2022). Discontinuation, suboptimal adherence, and reinitiation of oral HIV pre-exposure prophylaxis: a global systematic review and meta-analysis. Lancet HIV.

[27] Ramautarsing R.A., Meksena R., Sungsing T. (2020). Evaluation of a pre-exposure prophylaxis programme for men who have sex with men and transgender women in Thailand: learning through the HIV prevention cascade lens. J Int AIDS Soc.

[28] Hall A.K., Cole-Lewis H., Bernhardt J.M. (2015). Mobile text messaging for health: a systematic review of reviews. Annu Rev Public Health.

[29] Liu A.Y., Vittinghoff E., von Felten P. (2018). Randomized controlled trial of a mobile health intervention to promote retention and adherence to preexposure prophylaxis among young people at risk for human immunodeficiency virus: the EPIC study. Clin Infect Dis.

[30] Serrano V.B., Moore D.J., Morris S. (2023). Efficacy of daily text messaging to support adherence to HIV pre-exposure prophylaxis (PrEP) among stimulant-using men who have sex with men. Subst Use Misuse.

[31] van den Elshout M.A.M., Hoornenborg E., Achterbergh R.C.A. (2021). Improving adherence to daily preexposure prophylaxis among MSM in Amsterdam by providing feedback via a mobile application. AIDS.

[32] Moore D.J., Jain S., Dubé M.P. (2018). Randomized controlled trial of daily text messages to support adherence to preexposure prophylaxis in individuals at risk for human immunodeficiency virus: the TAPIR study. Clin Infect Dis.

[33] Kawichai S., Songtaweesin W.N., Wongharn P. (2022). A mobile phone app to support adherence to daily HIV pre-exposure prophylaxis engagement among young men who have sex with men and transgender women aged 15 to 19 Years in Thailand: pilot randomized controlled trial. JMIR Mhealth Uhealth.

[34] Chautrakarn S., Rayanakorn A., Intawong K. (2022). PrEP stigma among current and non-current PrEP users in Thailand: a comparison between hospital and key population-led health service settings. Front Public Health.

[35] Pengnonyang S., Ramautarsing R.A., Janyam S. (2022). Certification of lay providers to deliver key population-led HIV services in Thailand's National Healthcare System: lessons learned. J Int AIDS Soc.

[36] McClure C., Chandler C., Bissell S. (2015). Responses to HIV in sexually exploited children or adolescents who sell sex. Lancet.

[37] Platt L., Grenfell P., Fletcher A. (2013). Systematic review examining differences in HIV, sexually transmitted infections and health-related harms between migrant and non-migrant female sex workers. Sex Transm Infect.

