# Supplementary material for: High PrEP uptake, adherence, persistence and effectiveness outcomes among young Thai men and transgender women who sell sex in Bangkok and Pattaya, Thailand: findings from the open-label combination HIV prevention effectiveness (COPE) study
Source: Lancet Reg Health Southeast Asia. 2023 May 25;15:100217. doi: 10.1016/j.lansea.2023.100217 (PMC10442968; doi:10.1016/j.lansea.2023.100217)
Supplement: Supplemental Files S1–S6 [file mmc1.docx]

High PrEP Uptake, Adherence, Persistence and Effectiveness Outcomes Among Young Thai Men and Transgender Women Who Sell Sex in Bangkok and Pattaya, Thailand: Findings from the Open-Label Combination HIV Prevention Effectiveness (COPE) Study

Brian W. Weir^1,2^, Andrea L. Wirtz^1^, Tareerat Chemnasiri^3,4^, Stefan D. Baral^1^, Michele Decker^1,5^, Chen Dun^1,2^, Sandra Hsu Hnin Mon^1^, Chaiwat Ungsedhapand ^3,4^, Eileen F. Dunne^3,4^, Joseph Woodring^3,4^, Sarika Pattanasin^3,4^, Wichuda Sukwicha^3,4^, Michael C. Thigpen^3,4^, Anchalee Varangrat^3,4^, Anchalee Warapornmongkholkul^3,4^, Siobhan O’Connor^3^, Julie P. Ngo^1,6^, Noor Qaragholi^1^, Haley I. Sisel^1,6^, Jasmine M. Truong^1^, Surang Janyam^7^, Danai Linjongrat^8^, Somchai Sriplienchan^7^, Pachara Sirivongrangson^4^, James F. Rooney^9^, Patrick Sullivan^10^, Boosbun Chua-Intra^4^, Andrew C. Hickey^3,4^, & Chris Beyrer^1^ on behalf of the COPE Study Team

^1^ Center for Public Health and Human Rights, Department of Epidemiology, Johns Hopkins Bloomberg School of Public Health, Baltimore, MD, USA

^2^ Department of Health, Behavior & Society, Johns Hopkins Bloomberg School of Public Health, Baltimore, MD, USA

^3^ Division of HIV Prevention, U.S. Centers for Disease Control and Prevention, Atlanta, GA, USA

^4^ Division of HIV Prevention, Thailand Ministry of Public Health-U.S. Centers for Disease Control and Prevention Collaboration, Nonthaburi, Thailand

^5^ Department of Population, Family & Reproductive Health, Johns Hopkins Bloomberg School of Public Health, Baltimore, MD, USA

^6^ Department of International Health, Johns Hopkins Bloomberg School of Public Health, Baltimore, MD, USA

^7^ Service Workers in Group Foundation (SWING), Bangkok and Pattaya, Thailand

^8^ Rainbow Sky Association of Thailand (RSAT), Bangkok, Thailand

^9^ Medical Affairs, Gilead Sciences, Foster City, CA, USA

^10^ Department of Epidemiology, Emory University Rollins School of Public Health, Atlanta, GA, USA

^11^ Division of AIDS, National Institute of Allergy and Infectious Diseases, National Institutes of Health, Bethesda, MD, USA

^12^ Department of Epidemiology, Mahidol University, Nakhon Pathom, Thailand

^13^ Asia Pacific Coalition on Male Sexual Health Foundation (APCOM), Bangkok, Thailand

**Contents:**

[**Supplemental File 1.** Study flow diagram and participant study visits for the Combination Prevention Effectiveness (COPE) study for young men who have sex with men and transgender women who exchange sex, Thailand (2018 – 2020) 3](#_Toc133476436)

[**Supplemental File 2.** Participant compensation schedule for the Combination Prevention Effectiveness (COPE) study for young men who have sex with men and transgender women who exchange sex, Thailand (2018 – 2020) 5](#_Toc133476437)

[**Supplemental File 3.** Validation of self-reported PrEP adherences using dried blood spots: methods and results 6](#_Toc133476438)

[**Supplemental File 4.** Sensitivity analysis of HIV incidence based on a randomly select a date of HIV infection between the last negative HIV test and first positive HIV test: methods and results 7](#_Toc133476439)

[**Supplemental File 5.** Participant follow-up in the Combination Prevention Effectiveness (COPE) study for young men who have sex with men and transgender women who exchange sex, Thailand (2018 – 2020) 8](#_Toc133476440)

[**Supplemental File 6.** Author contributions 9](#_Toc133476441)

# **Supplemental File 1.** Study flow diagram and participant study visits for the Combination Prevention Effectiveness (COPE) study for young men who have sex with men and transgender women who exchange sex, Thailand (2018 – 2020)


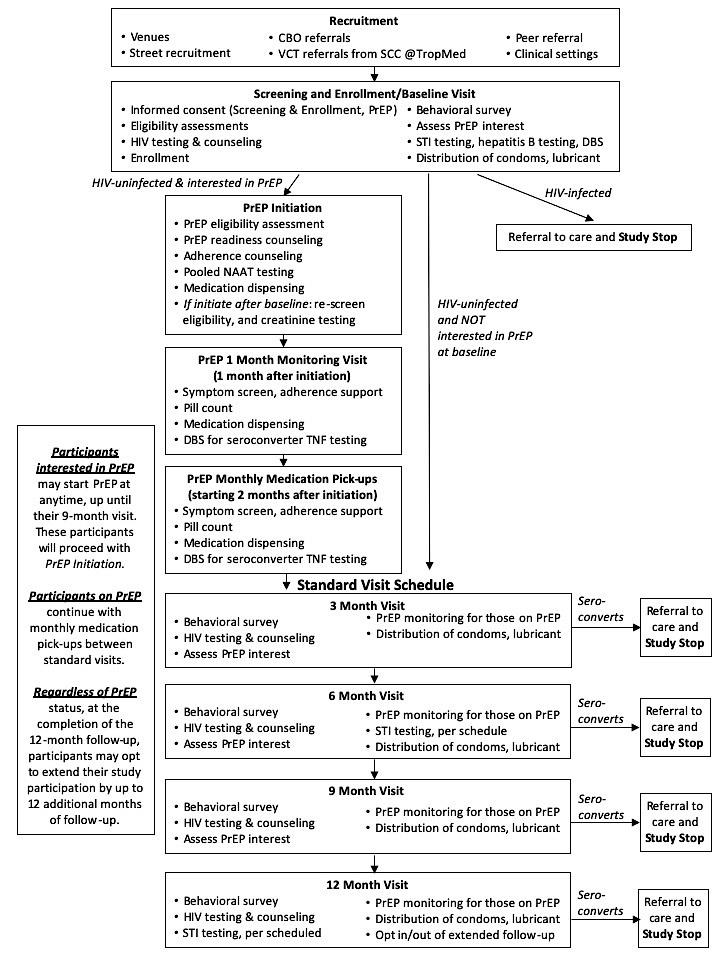


Footnote: CBO= community-based organization; VCT= voluntary counseling and testing; PrEP= pre-exposure prophylaxis; DBS= dried blood spots; NAAT= nucleic acid amplification test

# **Supplemental File 2.** Participant compensation schedule for the Combination Prevention Effectiveness (COPE) study for young men who have sex with men and transgender women who exchange sex, Thailand (2018 – 2020)

| Study component | THB | $ US |
| --- | --- | --- |
| Eligibility screening and ineligible for study | 100 | 35.00 |
| Baseline study visit | 1000 | 35.00 |
| Quarterly study visits | 1000 | 35.00 |
| Additional courtesy award for 12-month study visit | 500 | 17.50 |
| Additional courtesy award for 24-month study visit | 500 | 17.50 |
| Weekly SMS survey completion | 30 | 0.83 |
| Courtesy award for completion of 4 SMS surveys in month | 100 | 3.50 |
| PrEP medication pick-up visit | 500 | 17.50 |
| Acute HIV infection symptoms visit while taking PrEP | 500 | 17.50 |

Note: THB = Thai baht; $ US = United States dollars. See protocol paper^1^ for narrative description of the compensation schedule.

# **Supplemental File 3.** Validation of self-reported PrEP adherences using dried blood spots: methods and results

*Methods:* Self-reported PrEP use and adherence were validated with a sub-sample analysis of TFV-DP levels in dried blood spots (DBS) from quarterly assessments. DBS from 30 participants who completed three, six, nine, and 12-month assessments were randomly selected, including ten participants who reported no PrEP use across all four quarterly assessments; ten participants who reported poor adherence at one or more assessments; and ten participants who reported good adherence. TFV-DP concentrations above 700fmol/3mm punch were considered indicative of good adherence. ^2^

*Results:* In the subsample of DBS from participants with self-reported adherent use, 53 of 66 (80·3%) had sufficient TFV-DP levels (>700 fmol/3mm punch), seven (10·6%) had detectable but insufficient TFV-DP, and six (9·1%) had no detectable TFV-DP (< 200 fmol/3mm punch). Of seven DBS samples with self-reported insufficient PrEP adherence, three (42·9%) had sufficient TFV-DP levels, two (28·6%) had insufficient TFV-DP levels, and two (28·6%) had no detectable TFV-DP. Of the 47 samples with no self-reported PrEP, one (2·1%) had sufficient TFV-DP levels, three (6·4%) had insufficient TFV-DP levels, and 43 (91·5%) had no detectable TFV-DP. The overall kappa statistic indicated very good agreement between PrEP adherence based on self-reported and PrEP adherence based on TFV-DP levels (81·7%; *χ*^2^ = 82·6, p <0·001). ^3^

*References:*

1. Wirtz AL, Weir BW, Mon SHH, et al. Testing the Effectiveness and Cost-Effectiveness of a Combination HIV Prevention Intervention Among Young Cisgender Men Who Have Sex With Men and Transgender Women Who Sell or Exchange Sex in Thailand: Protocol for the Combination Prevention Effectiveness Study. *JMIR Res Protoc* 2020; **9**(1): e15354-e.

2. Deutsch MB, Glidden DV, Sevelius J, et al. HIV pre-exposure prophylaxis in transgender women: a subgroup analysis of the iPrEx trial. *Lancet HIV* 2015; **2**(12): e512-9.

3. Byrt T. How good is that agreement? *Epidemiology* 1996; **7**(5): 561.

# **Supplemental File 4.** Sensitivity analysis of HIV incidence based on a randomly select a date of HIV infection between the last negative HIV test and first positive HIV test: methods and results

*Methods:* We conducted a sensitivity analysis of HIV incidence using multiple imputations to randomly select a date of HIV infection between the last negative HIV test and first positive HIV test. We generated 10,000 data sets in which the date of seroconversion for each HIV infection was randomly selected from a uniform distribution. For each imputed dataset, the incidence rates and incidence rate ratio (IRR) with exact confidence intervals and exact p-values were calculated.

*Results:* Among the 10,000 imputed datasets with day of infection randomly selected for the ten participants with seroconversion, 86·1% had one or fewer infections during PrEP use (p < 0·001), 97·9% had two or fewer infections during PrEP use (p < 0·005), and 99·9% had three or fewer infections during PrEP use (p < 0·05). Only 0·13% imputed data sets had four infections during PrEP use (p = 0·068), and none had five infections during PrEP use. Based on the imputations, the estimated IRR of HIV infection for time on PrEP vs. time not on PrEP was 0·041 (p < 0·001; 95% CI = 0·006 – 0·311).

# **Supplemental File 5.** Participant follow-up in the Combination Prevention Effectiveness (COPE) study for young men who have sex with men and transgender women who exchange sex, Thailand (2018 – 2020)

# **Supplemental File 6.** Author contributions
