# Supplementary material for: High PrEP uptake, adherence, persistence and effectiveness outcomes among young Thai men and transgender women who sell sex in Bangkok and Pattaya, Thailand: findings from the open-label combination HIV prevention effectiveness (COPE) study
Source: Lancet Reg Health Southeast Asia. 2023 May 25;15:100217. doi: 10.1016/j.lansea.2023.100217 (PMC10442968; doi:10.1016/j.lansea.2023.100217)
Supplement: Translated abstract in Thai [file mmc2.docx]

*Disclaimer: This translation in Thai was submitted by the authors and we reproduce it as supplied. It has not been peer reviewed. Our editorial processes have only been applied to the original abstract in English, which should serve as reference for this manuscript.*

บทย่อ

ความเป็นมา

ยากินทุกวันเพื่อป้องกันการติดเชื้อก่อนการสัมผัสเชื้อ (เพร็พ) เป็นการป้องกันการติดเชื้อเอชไอวีที่มีประสิทธิภาพ แต่ไม่มีการศึกษาใดที่ประเมินมาตรการป้องกันเอชไอวีแบบผสมผสานด้วยการให้เพร็พสำหรับสาวประเภทสองและชายที่มีเพศสัมพันธ์กับชายที่ขายบริการทางเพศ

วิธีการศึกษา

การศึกษาประสิทธิผลของมาตรการป้องกันแบบผสมผสาน (โคป) เป็นการศึกษาระดับชุมชนโดยไม่มีการสุ่มเลือกประชากรในกรุงเทพฯ และพัทยา ประเทศไทย ประชากรที่ศึกษาคือชายที่มีเพศสัมพันธ์กับชายและสาวประเภทสองอายุ 18-26 ปีที่ไม่ติดเชื้อเอชไอวี ซึ่งรายงานว่ามีการแลกเปลี่ยนบริการทางเพศกับชายในช่วง 12 เดือนที่ผ่านมา และมีคุณสมบัติตรงตามเกณฑ์การรับเพร็พของบริการสาธารณสุขสหรัฐอเมริกาปี 2014 มาตรการป้องกันแบบผสมผสานประกอบด้วยการตรวจหาเชื้อเอชไอวีทุกสามเดือน ตรวจหาโรคติดต่อทางเพศสัมพันธ์ทุกหกเดือน ให้ถุงยางอนามัยและสารหล่อลื่น และเสนอให้เริ่มหรือหยุดกินเพร็พได้ตลอดเวลาระหว่างเข้าร่วมการวิจัย ผู้เข้าร่วมการวิจัยที่กินเพร็พได้รับการให้คำปรึกษาด้านวินัยในการกินยาทุกเดือนและข้อความสั้นเตือนให้กินยา ผลการศึกษาหลักคืออัตราส่วนอุบัติการณ์การติดเชื้อเอชไอวีต่อการกินเพร็พเปรียบเทียบกับการไม่กินเพร็พ ผลการศึกษารองคือการเริ่มกินเพร็พ การกินเพร็พใน 12 เดือน และวินัยในการกินเพร็พ

ผลการศึกษา

ตั้งแต่เดือนตุลาคม 2017 ถึงเดือนสิงหาคม 2019 มี**ผู้เข้าร่วมวิจัยจำนวน 846 คน**เข้าร่วมการศึกษา โดยมี**ผู้เข้าร่วมวิจัยจำนวน** 531 คน (62.8%) เริ่มกินเพร็พทันที **ผู้เข้าร่วมวิจัยจำนวน** 104 คน (12.3%) เริ่มกินเพร็พหลังจากนั้น และ**ผู้เข้าร่วมวิจัยจำนวน 211 (24.9%) เลือกไม่กินเพร็พ ในกลุ่มผู้เข้าร่วมวิจัยที่เริ่มกินเพร็พภายใน 30 วันหลังจากเข้าร่วมการวิจัย พบว่า 85.9% กินเพร็พทั้ง 12 เดือน เมื่อกินเพร็พ ผู้เข้าร่วมวิจัยรายงานวินัยการกินเพร็พในการประเมินทุกสามเดือนที่ 94.9% มีผู้เข้าร่วมวิจัยที่ไม่ได้กินเพร็พจำนวน 10 คนติดเชื้อเอชไอวี (อัตราอุบัติการณ์ [R] = 3.42 ต่อ 100 คน-ปี [PY]; 95%CI = 1.64-6.30) ในขณะที่ไม่มีผู้เข้าร่วมวิจัยที่กินเพร็พติดเชื้อเอชไอวี (IR = 0.0 ต่อ 100 คน-ปี [PY]; 95%CI = 0.0-0.62) โดยที่ IRR = 0.0 (95%CI = 0.0-0.22; p<0.001)**

**การแปลผล**

เยาวชนไทยที่เป็นชายที่มีเพศสัมพันธ์กับชายและสาวประเภทสองที่ขายบริการทางเพศสามารถกินเพร็พได้ในระดับสูง สามารถกินเพร็พอย่างต่อเนื่องและมีวินัยในการกินยาในระดับสูง และอุบัติการณ์การติดเชื้อเอชไอวีต่ำเมื่ออยู่ในสภาพแวดล้อมที่มีการสนับสนุนจากชุมชน

**ทุนการวิจัย**

สถาบันโรคภูมิแพ้และโรคติดเชื้อแห่งชาติสหรัฐอเมริกา ศูนย์ควบคุมและป้องกันโรคแห่งสหรัฐอเมริกา
