# Supplementary material for: High PrEP uptake, adherence, persistence and effectiveness outcomes among young Thai men and transgender women who sell sex in Bangkok and Pattaya, Thailand: findings from the open-label combination HIV prevention effectiveness (COPE) study
Source: Lancet Reg Health Southeast Asia. 2023 May 25;15:100217. doi: 10.1016/j.lansea.2023.100217 (PMC10442968; doi:10.1016/j.lansea.2023.100217)
Supplement: Beyrer_COPE Study_Study Group for Indexing [file mmc3.docx]

**COPE Study Team**

| **First** | **Name** |
| --- | --- |
| Chris | Beyrer |
| Andrea | Wirtz |
| Brian | Weir |
| Stefan | Baral |
| Michele | Decker |
| Sandra Hsu Hnin | Mon |
| James | Case |
| Chen | Dun |
| Jasmine | Truong |
| Noor | Qaragholi |
| Julie | Ngo |
| Haley | Sisel |
| Pachara | Sirivongrangson |
| Boosbun | Chua-Intra |
| Anupong | Chitwarakorn |
| Wasin | Matsee |
| Pratakpong | Wongkiti |
| Chidanan | Krasan |
| Anchana | Chainuwong |
| Nauwarat | Imlimtharn |
| Potcharawan | Reansoi |
| Teeraparp | Watanatanyaporn |
| Jarupa | Nuamlert |
| Supannikar | Namwong |
| Jutarat | Phetnark |
| Wachirawit | Supasa |
| Siriporn | Sueayot |
| Andrew | Hickey |
| Michael | Thigpen |
| Eileen | Dunne |
| Joseph | Woodring |
| Christie | Vu |
| Siobhan | O’Connor |
| Patrick | Flaherty |
| Timothy | Holtz |
| Tareerat | Chemnasiri |
| Anchalee | Varangrat |
| Anchalee | Warapornmongkholkul |
| Anekpong | Chanthaweesirirat |
| Warunee | Thienkrua |
| Wichuda | Sukwicha |
| Pitthaya | Disprayoon |
| Kanjana | Kamkong |
| Dararat | Worrajittanon |
| Supawadee | Na-Pompet |
| Chonlanot | Sariwatta |
| Patnaree | Oungprasertgul |
| Phanurassamee | Sittidech |
| Jirawat | Suksamosorn |
| Kesinee | Sujina |
| Chaiwat | Ungsedhapand |
| Wannee | Chonwattana |
| Nichnawee | Kamchaithep |
| Sarika | Pattanasin |
| Nongkran | Tatakham |
| Pikunchai | Luechai |
| Philip | Mock |
| Betsy | Cadwell |
| Ram | Shrestha |
| Baranee | Balmongkol |
| Boonyos | Raengsakulrach |
| Wanna | Leelawiwat |
| Wanna | Suwannaphan |
| Achara | Sriinsut |
| Punneeporn | Wasinrapee |
| Pornchanok | Chanathalay |
| Nutthawoot | Promda |
| Santi | Winaitham |
| Oranuch | Kongpechsatit |
| Kusuma | Auethavornanan |
| Jaray | Tongtoyai |
| Pairote | Tararut |
| Atitaya | McNamara |
| Famui | Mueanpai |
| Natthaga | Sakulploy |
| Kanokpan | Pancharoen |
| Chariya | Utenpitak |
| Caroline | Fukuda |
| Thitima | Cherdtrakulkiat |
| Tanyawarin | Janthiraj |
| Anuwat | Sriporn |
| Natee | Prathummart |
| Patsaraporn | Khongsom |
| Navakarn | Navanuch |
| Rinda | Wongbenchaporn |
| Chanya | Peerapatdit |
| Pechpailin | Khlaimanee |
| Patcharat | Niyamakom |
| Narongritt | Tippanont |
| Somsak | Yafant |
| Tatchai | Ruanpang |
| Siripak | Pongthai |
| Kamolnetr | Okanurak |
| Aronrag | Meeyai |
| Danai | Linjongrat |
| Phubet | Panpet |
| Orawan | Fungfoosri |
| Prisana | Boonyawan |
| Theeranat | Sangprasert |
| Natthawirojn | Inthanin |
| Teppanan | Sangiamjit |
| Somporn | Saiwaew |
| Konlawat | Pawong |
| Surang | Janyam |
| Chamrong | Phaengnongyang |
| Atachai | Phunkron |
| Denchai | Srikrongthong |
| Thanaphat | Dokrak |
| Phathranis | Meekrua |
| Saman | Sumalu |
| Cawee | Kanlose |
| Prasopsuk | Thapwong |
| Kritsanapol | Kaewboonta |
| Pornpichit | Brutrat |
| Waris | Watthanayeam |
| Apichat | Udomjirasirichot |
| Somchai | Sriplienchan |
| Midnight | Poonkasetwattana |
| Silapakhon | Kongsakul |
| Michael | Badorrek |
| Andrey | Tran |
| Ryan | Figueiredo |
| Safir | Soeparna |
| Wattana | Keiangpa |
| Apiwit | Tibamrung |
| Sunadda | Samana |
| Hidayah | Syahputra |
| Worapon | Rattanawarawong |
| Patrick | Sullivan |
| Rachel | Valencia |
| Usha | Sharma |
| Adeola | Adeyeye |
| James | Rooney |
| Pojjana | Hunchangsith |
| Tanyaporn | Wansom |
| Thomas | Guadamuz |
| Annette | Sohn |
